# Dendritic Cells and SARS-CoV-2 Infection: Still an Unclarified Connection

**DOI:** 10.3390/cells9092046

**Published:** 2020-09-08

**Authors:** Pasquale Campana, Valentina Parisi, Dario Leosco, Debora Bencivenga, Fulvio Della Ragione, Adriana Borriello

**Affiliations:** 1 Department of Translational Medical Sciences, University of Naples ‘Federico II’, Via Sergio Pansini 5, 80131 Naples, Italy; pasq.campana@unina.it (P.C.); valentina.parisi@unina.it (V.P.); dleosco@unina.it (D.L.); 2 Department of Precision Medicine, University of Campania “L. Vanvitelli”, Via De Crecchio 7, 80138 Naples, Italy; debora.bencivenga@unicampania.it

**Keywords:** SARS-CoV-2, Covid-19, spike protein, dendritic cells, DC-SIGN

## Abstract

The ongoing pandemic due to Severe Acute Respiratory Syndrome Coronavirus 2 (SARS-CoV-2) has so far infected about 2.42 × 10^7^ (as at 27 August 2020) subjects with more than 820,000 deaths. It is the third zoonotic coronavirus-dependent outbreak in the last twenty years and represents a major infective threat for public health worldwide. A main aspect of the infection, in analogy to other viral infections, is the so-called “cytokine storm”, an inappropriate molecular response to virus spread which plays major roles in tissue and organ damage. Immunological therapies, including vaccines and humanized monoclonal antibodies, have been proposed as major strategies for prevention and treatment of the disease. Accordingly, a detailed mechanistic knowledge of the molecular events with which the virus infects cells and induces an immunological response appears necessary. In this review, we will report details of the initial process of SARS-CoV-2 cellular entry with major emphasis on the maturation of the spike protein. Then, a particular focus will be devoted to describe the possible mechanisms by which dendritic cells, a major cellular component of innate and adaptive immune responses, may play a role in the spread of the virus in the human body and in the clinical evolution of the disease.

## 1. Introduction

The first report describing a human infection due to a coronavirus was published in 1965 in the British Journal of Medicine [1]. The authors, Tyrrell and Bynoe, described the isolation and growth in organ cultures (mainly from human embryo tracheas) of a novel common cold virus distinct from all the known viruses. A few years later, in 1968, Tyrrell and colleagues identified and started to characterize the family to which it belongs [2]. The term coronaviruses was coined based on its microscopic appearance, that is, spherical particles with projections that emanate from the surface like a crown. Since then, it has become evident that numerous different members of the Coronaviridae family circulate in various mammals and birds, as well as in the human population. Initially, it seemed that they only cause minor diseases of the respiratory tract in humans. However, the first unexpected outbreak occurred in 2002, due to a coronavirus infection [3]. About ten years later, a second coronavirus-associated pandemic arose, which, although characterized by a high mortality rate, was fortunately scarcely contagious [3]. These two outbreaks should now be considered simply the tip of an iceberg made of coronavirus-dependent diseases that, with different lethality, affect numerous animal hosts. In fact, veterinary coronavirus experts have already warned of the potential virulence of these infectious agents in causing fatal respiratory or enteric diseases [4]. Moreover, new coronavirus strains have suddenly and continuously emerged causing important diseases like the porcine epidemic diarrhea CoV (PEDV) [5]. Generally speaking, the world of viruses (the so-called virosphere) is scarcely known, although the role of viruses in host regulation/evolution and in the maintenance of natural ecosystems has become increasingly accepted. Noteworthy, it has been estimated that several hundred thousand different viruses exist and they encode a huge number of proteins, which are mostly absent in the available protein databases and perform completely unknown functions. This, in turn, suggests that the functional interactions identified between eukaryotic cells and viruses (including coronaviruses) are very limited. In addition, an understanding of the possible genetic origin and evolution of the viral envelope components that recognize proteins on the human cell surface and allow virus entry must be achieved. This is particularly necessary to develop strategies for the treatment, control, and prevention of potentially unexpected human outbreaks dependent on viruses.

In this review, we will analyze some aspects of the ongoing coronavirus outbreak due to an infective agent with a very remarkable spread potential, namely, Severe Acute Respiratory Syndrome Coronavirus 2 (SARS-CoV-2). We will describe molecular/functional aspects of its key envelope protein, the spike protein (forming the corona structure), and the virus putative interaction with the dendritic cells, a cell population that plays a fundamental role in the innate/adaptive immune response.

## 2. SARS-CoV-2, the Pathogen Responsible for Covid-19 Pandemic

Coronaviruses are enveloped positive-sense single-stranded RNA viruses. Their genome is the largest among all the RNA viruses (27.6–31 kb) [4]. This virus family includes four different genera, namely alpha-, beta-, gamma-, and delta-coronaviruses. Bats and rodents represent the common reservoir hosts for alpha and beta-coronaviruses, while birds for the other two genera. However, coronaviruses are able to jump from their physiological reservoirs to other animals. Humans can be infected, but the transmission from the animal reservoir to humans generally seems to require an intermediate host [4,6].

A total of seven coronaviruses able to infect humans have been reported [4]. Among them, three strongly pathogenic human beta-coronaviruses have been identified in the last two decades [6]. Between 2002 and 2004, the SARS-CoV infected about 8400 people, killing less than 800 patients with a fatality rate estimated around 10% [7]. In 2012, a second zoonotic outbreak associated with another beta-coronavirus appeared due to MERS-CoV (Middle East respiratory syndrome coronavirus), a highly lethal virus that, fortunately, was difficult to transmit (about 2500 cases with a mortality of 34.3%) [8]. The third zoonosis has been due to the SARS-CoV-2 described initially in China at the end of 2019. Differently from the previous two infections, the virus has rapidly spread globally causing a devastating pandemic and a very serious problem for worldwide health [9,10]. As at today (27 August 2020), more than 2.42 × 10^7^ total reported cases and 820,000 deaths have been counted (Coronavirus Resource Center, Johns Hopkins University of Medicine). The mortality rate (infection fatality rate) cannot be precisely established as the pandemic is still ongoing and the number of infected subjects is not exactly defined and certainly higher than the diagnosed infections.

In addition to the three highly pathogenic coronaviruses reported above, two coronaviruses (HCoV-229 and HCoV-OC43), indirectly derived from bats and rodents, were found in humans before SARS-CoV identification. Successively, after SARS-CoV identification but before MERS-CoV and SARS-CoV-2 recognition, HCoV-NL63 and HCoV-HKU were also characterized. They are all responsible for mild respiratory diseases. Given their large presence in different animal species (where, generally, they do not cause fatal diseases), coronaviruses have mainly been investigated by veterinarians. Importantly, they represent a paradigmatic example of virus evolution with high genetic variability and great potential for recombination. These features are expected to cause major changes in the biological characteristics of the virus and are important factors predisposing to novel pandemics [11]. The view is directly confirmed by the emergence of three outbreaks in just two decades due to novel human coronaviruses. A discussion about the bases and mechanisms that have produced three infective/lethal coronaviruses is out of the scope of the present review, but several lines of evidence suggest that their origin might probably be correlated to the negative human impact on the environment.

On the basis of available genome sequences, SARS-CoV-2 has been strongly related (about 92–96% identity) to BatCoVRaTG13, a coronavirus identified in a bat species (*Rhinolophus affinis*). It is also, but less closely, correlated to Pangolin-CoV isolated from pangolin lung (91%) [12]. Accordingly, it has been suggested, but not proved, that pangolin is the intermediary host in the Covid-19 pandemic. Finally, SARS-CoV-2 is identical to SARS-CoV for about 79.6% [13].

Although coronaviruses have a proofreading mechanism, massive and global spreading provides SARS-CoV-2 with a huge mutation opportunity (more than 10 SARS-CoV-2 mutants have been already characterized) as well as a high possibility of concomitant occurrence (i.e., in a single subject) of different coronavirus genomes for viral recombination [14]. So far, however, the major reported variation is D614G substitution that appears to increase transmission but not mortality [15,16]. The mutation occurred in only 10% of global sequences analyzed prior to March 1 and 78% of total sequences between April 1 and May 18, probably as a consequence of its high infectivity rate. The presence of novel virus forms and the high probability of recombination must be taken into consideration with the aim of eradicating the pandemic by means of vaccine(s) or antibody-based therapy.

**Figure 1:**
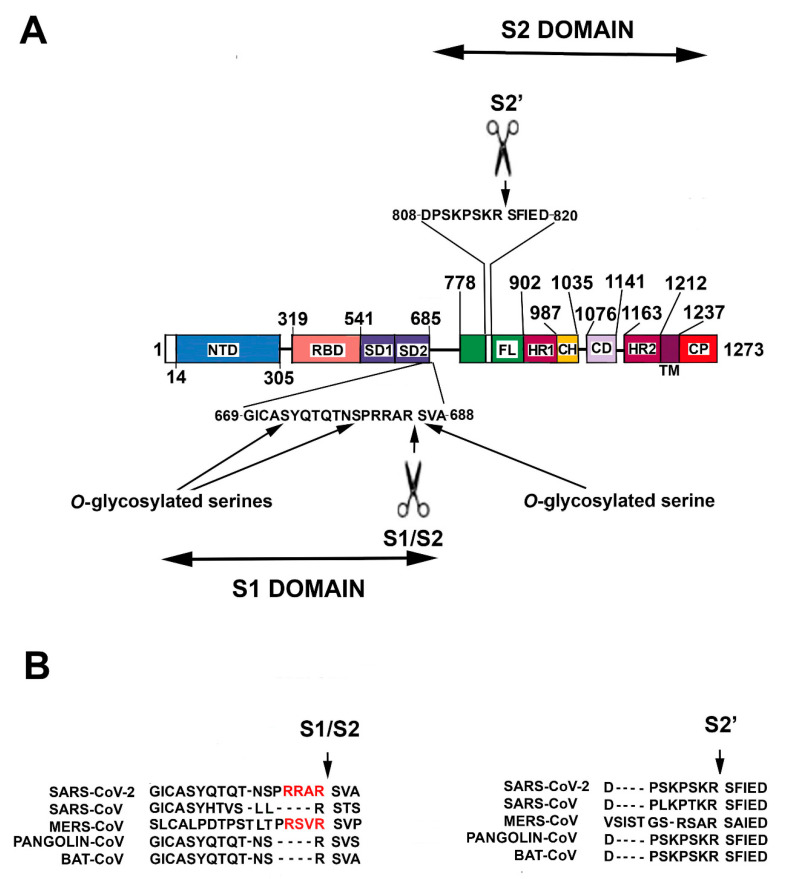
Domain organization and cleavage sequences of SARS-CoV-2 spike glycoprotein. (**A**) The domains of the protein are as the follows. S1 Domain: NTD (N-terminal domain), RBD (receptor binding domain), SD1 and SD2 (subdomain 1 and subdomain 2). S2 Domain: FL (fusion loop), HR1 (heptad repeat 1), CH (central helix), CD (connector domain), HR2 (heptad repeat 2), TM (trans-membrane domain), CP (cytoplasmic tail). The panel also reports the position of the residues at both the ends of the individual segments. (**B**) Consensus sequences for S1/S2 and S2′ cleavage sites. A comparison among SARS-CoV-2, SARS-CoV, MERS-CoV, Pangolin-CoV, and Bat-CoV is also reported. Red color highlights the polybasic sequence of SARS-CoV-2 and MERS-CoV (see text for details).

SARS–CoV-2 genome includes five major open reading frames encoding four structural proteins, namely nucleocapsidic (NP), membrane protein, envelope and spike (S) and a nonstructural replicase. NP is highly abundant in the virus where it stabilizes the coronavirus genome through direct viral RNA binding. Importantly, Covid-9 patients produce high levels of IgG against NP protein that might represent potential antigens in a vaccine setting. SARS-CoV-2 employs an envelope trimeric glycoprotein (S protein) to recognize the target cells. In brief, S protein includes two major functional domains, the N-terminal region (named S1) and a C-terminal region (S2) (Figure 1).

S2 plays a major role in the process of fusion between virus envelope and the membrane of the target cell that allows entry of the virus genetic material.

S1 contains a receptor binding domain (RBD) that is responsible for the recognition of, and binding to, its specific cell surface receptor, namely, angiotensin converting enzyme 2 (ACE2) [17,18]. Thus, to identify putatively the origin of SARS-CoV-2, it might be of interest to analyze the homology of S protein in various coronaviruses.

Structurally, S protein of SARS-CoV-2 is highly distinct from other CoV S proteins in that it shows less than 75% sequence identity to all described SARS-CoV. Conversely, it presents a 93.1% identity with respect to Bat-CoVRaTG13 [4,16]. Analyzing five critical amino acids in the RBD, a more complex condition is observed. Particularly, only one out of five critical RBD residues is maintained comparing SARS-CoV-2 and SARS-CoV or Bat-CoVRaTG13, while all five residues are conserved between SARS-CoV-2 and Pangolin-CoV. Therefore, it is tempting to hypothesize the occurrence of a recombination event(s) that inserts the Pangolin-CoV RBD into BatCoVRaTG13. To substantiate this view, however, the identification of SARS-CoV-2 animal reservoir is required.

As described before, an S1-independent S2 domain is required for viral entry into the host cell [17,18]. Thus, the cleavage of S protein at the S1/S2 site is absolutely required. Nonetheless, the precise knowledge of S protein activation still awaits a complete definition. Further insights into S protein cleavage are reported in the next paragraph.

## 3. Spike Protein Maturation and Cell Invasion

Key steps of SARS-CoV-2 infection are: (i) identification of target cells; (ii) S protein maturation and; finally, (iii) virus cell entry. Although all these phases have been extensively investigated, numerous uncertainties still exist and their solution is important in the elucidation of Covid-19 pathophysiology and development of therapeutic approaches.

SARS-CoV-2 S protein is a heavily glycosylated homotrimeric class I fusion protein of 1273 amino acid residues [19]. Class I fusion proteins allow membrane fusion revealing a central coiled-coil structure composed of a trimer of α-helical hairpins, after merging. The primary and archetypal example of homotrimeric class I fusion protein is the influenza virus hemagglutinin (HA) protein. Structurally, the SARS-CoV-2 S protein can be divided into a number of distinct structural and functional domains [19] (Figure 1). Starting from the N-end signal sequence, the N-terminal region (S1 region, aa 14–685) includes an N-terminal domain (aa 14–305), the RBD (aa 319–541, 193 residues), a subdomain 1 (SD1) and a subdomain 2 (SD2). Next, a protease cleavage sequence (S1/S2 cleavage site) separates S1 from S2, the following region, S1 represents the outer globular head of the protein, and S1 binding to its receptor favors S2 exposure. S2 domain contains (from the cleavage site to the C-terminal residue, aa 686–1273), a membrane fusion peptide, a second cleavage sequence (S2′ cleavage site), an internal membrane fusion peptide (IFP), two heptad repeat domains (HR1 aa 902–987 and HR2 aa 1163–1212), the transmembrane domain (aa 1213–1237) and a cytoplasmic domain (aa 1238–1273) [19]. The S2 region represents the stem of the protein and is inserted into the virus envelope. Importantly, the S protein occurs in two conformational states, a pre-fusion and a post-fusion state [17,18,19].

The initial step of the virus entry is the binding of the S protein to its cellular receptor that has been identified as ACE2, although other interactors are probable [20]. Using various experimental approaches (surface plasmon resonance experiments, pull-down assays and structural/mutagenesis analyses), SARS-CoV-2 RBD has been demonstrated to have higher affinity for human ACE2 compared to SARS-CoV RBD [21]. Additional experiments employing the entire S protein have shown that SARS-CoV spike protein binds ACE2 more strongly than SARS-CoV-2 spike [21].

The dynamic status of S protein might explain the apparent paradox. RBD exists in two states, namely, a standing-up state or a lying-down state. Only when RBD is in the standing-up state it binds the receptor. The SARS-CoV-2 spike protein is generally in the lying-down state, while SARS-CoV spike is in a standing-up state, thus, explaining the previous observation. In vivo, however, to keep RBD accessible for binding, SARS-CoV-2 spike is activated by a host protease, shifting towards a standing-up condition. This allows SARS-CoV-2 spike to have in vivo higher affinity for ACE2 than SARS-CoV.

After binding with ACE2, an initial (priming) cleavage event occurs between S1 and S2 domains. The S1/S2 cleavage site in SARS-CoV-2 S protein is distinct from the corresponding one in SARS-CoV, but it is similar to that in MERS-CoV [17,18,19] (Figure 1B). It presents multiple arginine residues (i.e., a multibasic Arg-Arg-Ala-Arg sequence, aa 682–685) that form an exposed loop [19]. The occurrence of a multibasic cleavage sequence appears of great relevance in increasing S activation. Indeed, it allows the specific sequence cleavage by several proteases, including furin [22,23,24]. Similarly, it has been seen in avian influenza A viruses that the presence of a multibasic cleavage sequence in HA protein is an important factor of virulence [23,24,25].

Subsequently to S1/S2 cut, S2′ cleavage site can be recognized and cut by the protease TMPRSS2 (transmembrane serine protease 2) as well as by PC1 (prohormone convertase 1), trypsin-like proteases and cathepsins. The proteolysis produces a mature S2 fusion protein able to allow virus entry. Conversely, viruses showing a monobasic cleavage site (like SARS-CoV) might only employ TMPRSS2 (and not furin or related proteases) for pre-activation and, thus, might mostly infect the respiratory tract whose cells are highly TMPRSS2-positive [18,26] (Figure 1B).

Furin is the best characterized member of the so-called PCSK (proprotein convertase subtilisin/kexin type) family. This protease is ubiquitously expressed except in muscle cells where low levels of furin are detectable [22]. The enzyme is synthesized as an inactive proenzyme and is integrated by its C-end transmembrane domain in endoplasmic reticulum (ER) membrane. After the removal of an inhibitory N-terminal domain during the transition from ER and Golgi apparatus, the protein is N-glycosylated. A large part of active furin accumulates in Golgi apparatus while a certain amount is transported to the cell surface. Then, from the cell surface it returns into the intracellular compartment via the endosomal pathway. In brief, furin is localized at the cell surface and in intracellular compartments and, thus, it might process both cytosolic and extracellular substrates [22]. The canonical sequence of furin cleavage is Arg-X-Lys/Arg-Arg↓ that is very similar with the multibasic sequence of SARS-CoV-2. The wide cellular expression of furin (and of proteases belonging to the same family) allows the pre-activation of the spike protein of SARS-CoV-2 on the surface of numerous ACE2-positive cell types, increasing virus transmissibility [22].

Moreover, abundant furin intracellular presence suggests that infected cells might release pre-activated virions into the extracellular microenvironment (namely with an already cleaved S1/S2 site). Indeed, immunoblotting analysis of VSV (vesicular stomatitis virus) pseudoparticles containing SARS-CoV-2 S protein showed, differently from pseudoparticles including SARS-CoV S, the occurrence of cleaved S [18,22]. The delivery of pre-activated viruses might be able to induce membrane fusion without a necessary ACE2 interaction. As a matter of fact, these pre-activated viruses might spontaneously (although slowly) mature S2 subunit, thus allowing membrane fusion and virus entry. Alternatively, the interaction with cells whose cellular membrane presents high TMPRSS2 (or cathepsin B/L) expression might accelerate S2 maturation and increase virus entry independently of ACE2 binding or possibly using alternative receptor [18,27].

In summary, SARS-CoV-2 S protein might be activated (having a multibasic cleavage site) by a number of proteases higher than that of SARS-CoV and particularly by the widely distributed furin. The virus can infect a high number of different cells and tissues, as demonstrated by the complex and still not completely clarified pathophysiology of Covid-19 disease. Moreover, SARS-CoV-2 shows an affinity for ACE2 higher than that of SARS-CoV and a mechanism of spike activation and cellular entry more similar with that of MERS-CoV rather than SARS-CoV.

An additional feature should also be considered, namely, the observation that a leading proline (proline 681) could allow the addition of *O*-glycan on serine 673, threonine 678 and serine 686, all residues localized near the S1/S2 cleavage site [22,23] (Figure 1A). Mechanistically, the addition of glycans might create a mucin-like protection that could safeguard the cleavage site and adversely affect the virulence. However, since the glycan moieties are generally added at the level of Golgi where furin is also localized, it is not clear whether the putative shield could really protect the cleavage site. A further effect of S1 binding on ACE2 is related to the possible reduction of the enzyme membrane levels and, in turn, to the consequence on the pathway regulated by the enzyme [28]. This topic will be discussed in a subsequent paragraph.

In conclusion, the process of SARS-CoV-2 S protein maturation still needs to be investigated in detail. It will give useful information not only for understanding the multifaceted aspects of the Covid-19 disease, but also the effects of new mutations of SARS-CoV-2 or other coronaviruses. A deeper knowledge of the importance of each S protein domain is also central to the development of effective SARS-CoV-2 vaccines.

## 4. Pyroptosis: A Key Step in Covid-19 Outbreak

As described in the previous paragraph, SARS-CoV-2 S protein allows viral entry by interacting with ACE2, an enzyme largely expressed on the surface of type 2 pneumocytes [29]. The high contagiousness of the virus might be directly explained by the elevated S1 affinity for ACE2 that is probably 10–20 higher than that of SARS-CoV S1 [30].

**Figure 2:**
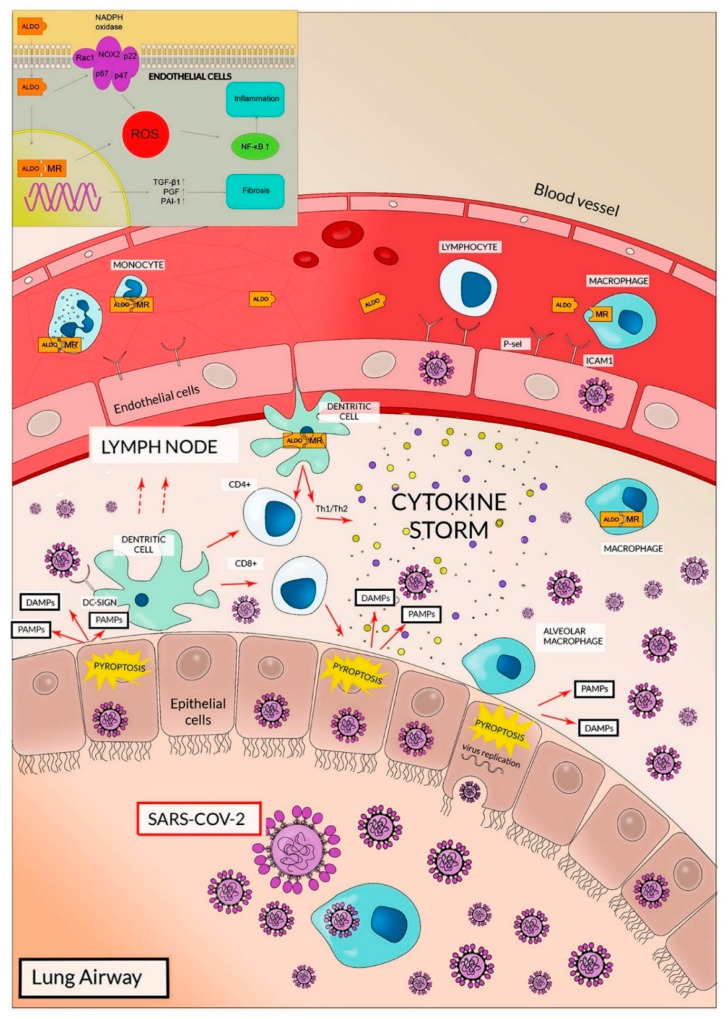
Scheme summarizing the possible involvement of dendritic cells in SARS-CoV-2 infection. Pneumocytes are infected by SARS-CoV-2 and, after pyroptosis, release viruses, PAMP (pathogen-associated molecular patterns) and DAMPS (damage-associated molecular patterns). PAMPs and DAMPs might activate dendritic cells. Moreover, DC-SIGN (localized on dendritic cells) might recognize and bind the virus. Then, dendritic cells might move toward lymph nodes. Locally, dendritic cells might affect different populations of T-lymphocytes. SARS-CoV-2 might also increase, through RAAS (renin-angiotensin-aldosterone system), the production of aldosterone (ALDO) that is intracellularly recognized by its receptor (MR) (see the text for more mechanistic details). In turn, this results in an activation of dendritic cells and macrophages. Aldosterone could also affect coagulation enhancing microthrombosis. SARS-CoV-2 also infects endothelial cells, causing hypercoagulation and affecting dendritic cells. The image also shows the cytokine storms that are due to the activation of a plethora of cells, including those of innate immunity response. In the inset, aldosterone stimulation of endothelial cells NADPH oxidases with a concomitant ROS production is shown. ROS might be also involved in the hyperinflammation condition.

The high expression of ACE2 and TMPRSS2 in lung cells clearly explains why, at least in the initial phases of the infection, the respiratory airways are involved in viral entrance/diffusion and in lung injury [18,26,29,31]. This also clarifies why the transmission of SARS-CoV-2 is predominantly through respiratory droplets, whereas the importance of oro-fecal transmission is not conclusively validated [6]. In lung airways, ACE2 expression has been reported, in addition to pneumocytes, on the cell surfaces of endothelial cells and alveolar macrophages that, in analogy with SARS-CoV infection, represent additional targets of SARS-CoV-2 (Figure 2) [29,32].

**Figure 3:**
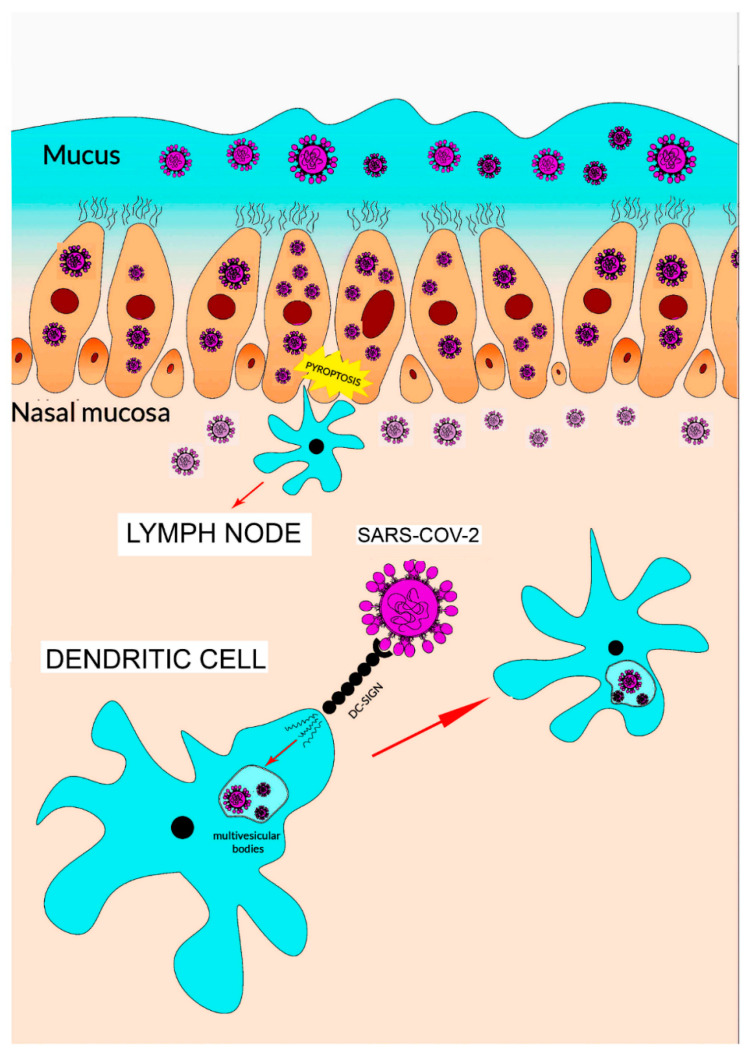
Scheme showing the putative dendritic cells involvement in SARS-CoV-2 entry through nasal epithelial cells. The entry of SARS-CoV-2 through epithelial cells is followed by pyroptosis and activation of dendritic cells in a manner similar with that reported in Figure 2. Particularly, the interaction between DC-SIGN and the virus is shown. The binding (and DAMP and PAMP interaction that is not shown) could cause the virus to spread from the nasal epithelial cells to the rest of human body.

*ACE2* and *TMPRSS2* genes are also co-expressed in nasal epithelial cells [27]. Accordingly, a detailed study suggests that this cell phenotype has a potential role in the initial phases of viral infection, spread and clearance (Figure 3).

After entry, viral replication induces a massive cell pyroptosis (Figure 2 and Figure 3) [6,33]. The term pyroptosis (from pyro, fire, and ptosis, falling) identifies a form of inflammatory programmed cell death pathway that, in humans, involves the activity of caspase-1, caspase-4 and caspase-5 (i.e., inflammatory caspases). These proteases are employed by the host cell to control infections by different pathogens like bacteria, viruses, fungi or protozoa. The process occurs through the activation of inflammatory caspases that cleave and activate the protein gasdermin D which, in turn, forms pores in the membrane. The following cell rupture allows the release of several molecules including cytokines like interleukin 1β (IL-1β) and interleukin 18 (IL-18). The released compounds activate a local cell response and an inflammatory reaction [34,35].

After cell invasion, viruses (including SARS-CoV-2) replicate and activate the formation of two intracellular complexes. One is defined as pathogen-associated molecular patterns (PAMPs) and the other is named damage-associated molecular patterns (DAMPs). PAMPs contain components derived from the specific infective pathogen that, in the case of viral infections, include single/double RNA/DNA, nucleocapsidic-derived and replication-derived compounds. DAMPs composition is more complex and heterogeneous; they are generally made of intracellular molecules (ATP, DNA, heat shock proteins) normally inaccessible to the immune system and released as a result of cell death/injury [6,33]. Other DAMP components might be portions of extracellular matrix molecule (hyaluronan fragments, heparin sulfate and others). The so-formed intracellular PAMPs and DAMPS activate a series of processes that result in the formation of inflammasome, the activation of inflammatory caspases, the formation of membrane pores and cell death [36]. To activate these phenomena, PAMPs and DAMPs need to be recognized by intracellular and extracellular receptors and, thus, induce intra and extracellular responses. The destruction of cells by pyroptosis results in the release of viruses, PAMPs, DAMPs and a variety of cytokines [34,35]. Thus, pyroptosis represents one of the initial steps of the so-called innate immune response, namely the initial host defense during infection. Innate immune system is the first actor in the initial recognition of a pathogen and in the following inflammatory response to reach an early block of the pathogen’s negative effects [36]. While the adaptive immune response shows a high degree of specificity due to the somatic recombination of lymphocytic genes encoding TCR and BCR (T- and B-cell receptors), the innate immunity is mostly due to phagocytic cells and to a variety of professional antigen-presenting cells including dendritic cells (DCs), macrophages and granulocytes. Innate immune response has been trivially considered non-specific. Conversely, PAMPs are specific for different classes of pathogens and their composition is peculiar for the infective agent [36]. Accordingly, different receptors have been evolved for identifying distinct types of PAMPs.

After the initial destruction of virus-infected cells by pyroptosis, cells of innate immune response bind PAMPs and DAMPs through an ample class of specific receptors (pattern-recognition receptors, PRRs) of which the Toll-like receptors (TLRs) are the most deeply characterized [36]. Specifically, TLRs2/4/7/8/9 recognize PAMPs derived from different viral components. Intracellular PRRs also exist and recognize intracellular PAMPs. In the case of viruses, PRRs are RIG-1 (retinoic acid-inducible gene 1), MDA5 (melanoma differentiation-associated protein 5) and NALP3 (NACHT, LRR and PYD domains-containing protein 3) and are responsible for inflammasome formation [36,37]. In particular, RIG-1 is a PRR important for recognizing cells that have been infected with viruses, including influenza A, West Nile virus, Sendai virus, Japanese encephalitis virus, flavivirus, and, importantly, coronaviruses [34,35,36,37,38,39].

TLR activation induces a variety of responses including the production of tumor necrosis factor (TNF), interleukin 6 (IL-6), type 1 IFN, monocyte chemoattractant 1 (MCP1), macrophage inflammatory protein 1 alpha and beta (MIP1alpha, MIP1beta) and interferon gamma-induced protein 10 (IP-10) [6,33]. In Covid-19, the innate immune system hyperactivity stimulates the acute lung injury (ALI) through cytokine release during the first phase of SARS-CoV-2 infection.

The high level of IL-1β (due to pyroptotic cell death) is also the major mechanism involved in migration of neutrophils and activation of T-cells [40,41]. As a matter of fact, a high concentration of neutrophils has been identified in lungs during Covid-19 pneumonia with a reduction of their blood levels. The activation of neutrophils cytotoxicity also induces ALI through the release of leukotrienes, reactive oxygen species (ROS) and, thus, this process might be considered as a fundamental contributor to the so-called cytokine storm. Neutrophils may also promote endothelial injury that plays a key role in viral systemic dissemination being also endothelial cells a privileged SARS-CoV-2 target [28,33,37,41].

The role of cytokine storms in the clinical features of Covid-19 pneumonia has also been demonstrated by high plasma levels of IL-6, IL-7, IL-10, TNF, granulocyte colony-stimulating factor and MIP 1α. In particular, the remarkable levels of IL-6 have shown their detrimental effects in amplifying the innate response and cytokine storm. It also correlates with the risk of mechanical ventilation and mortality in hospitalized Covid-19 patients [42,43]. In these subjects, the overproduction of IL-6 is mostly associated to macrophages and DCs, underscoring the role of innate immunity response [44,45,46,47].

## 5. Dendritic Cells and Lung Innate and Adaptive Immune Response

DCs are professional antigen-presenting cells and represent either the key components of innate response to pathogen infection or the orchestrators of the subsequent adaptive immunity. Three DC subtypes occur in the lung, namely type 1 and type 2 myeloid/conventional DCs (named cDC1 and cDC2 in mice) and plasmacytoid DC (pDCs) [48]. In humans, cDC1 population corresponds to CD141+ and cDC2 to CD1c+. CD141+ are present in the mucosa and vascular wall and, differently from other DC populations, are able to activate Th1 responses. CD1c+ are found in the lamina propria and are the major source of proinflammatory chemokines necessary for the recruitment of different inflammatory cells. This subtype also shows tolerogenic properties [48]. Finally, pDCs are distributed in all lung compartments, including the parenchyma, alveolar septa and airways. pDCs are essential for viral response in that their major physiological function is type I interferon production. In addition to these subtypes, DCs can also arise from circulating monocytes (Mo-DC) during inflammation [48,49].

After PAMP/DAMP interaction and TLR activation, immature DCs (iDCs) become mature DCs (mDCs) and migrate to lymph nodes for activating naive T cells. iDCs, and, although with a minor efficiency, mDCs, internalize, process and present captured antigens on class I (MHCI) and class II MHC molecules (MHCII). They distinguish between self- and foreign antigens using TLR [48]. Intriguingly, the migration to lymph nodes represents an important difference with macrophages that mostly play a tissue inherent antiviral activity. In SARS-CoV-2 infection, this is mainly exerted in the lung. In addition, part of mDC activity is played in the lung itself where these cells stimulate lung lymphoid follicles (LLF) containing classical germinal centers [50].

During their migration, DCs undergo phenotypical and functional maturation. Particularly, they up-regulate the expression of co-stimulatory molecules (CD80 and CD86) and the chemokine receptor CCR7. In addition, DCs secrete pro-inflammatory cytokines such as TNF-α and IL-12. After reaching the lymph node subcapsular sinus, DCs move to T-cell zones. Here, DCs are actively implicated in the antigen presentation to T cells. DCs have a very high capability of stimulating T-lymphocyte response [45,48]. It has been proposed that one DC is able to activate 100–3000 T cells. In lymph nodes, DCs stimulate, by presenting a specific antigen alongside the costimulatory signal, the activation of several T populations, including helper T-cells (CD4+ T-cells), cytotoxic T lymphocyte (CD8+ T-cells) and B-cells. Under specific conditions, DCs play a role in immune tolerance [51]. Importantly, DCs are the most potent antigen-presenting cells (the other are macrophages and B-cells) and the only able to activate antigen-specific naive CD8+ T cells [48]

## 6. Coronavirus Infection and Dendritic Cells

A complex and broad spectrum of clinical manifestation has been associated to the Covid-19 disease. Although a plethora of detailed (sometimes contradictory) molecular/clinical descriptions are available, several pathogenetic aspects are still elusive and await further mechanistic investigation. The variable severity of symptoms is likely due to a variety of factors which include not only age, genetic background and health status of the patient, but also environmental conditions, pollution and health system organization. Thus, the contribution of each factor should await more statistically significant studies.

On the other hand, the critical hyperinflammatory response, the multiorgan failure and the importance of comorbidities suggest a complexity of host-spreading mechanisms and cell target identification far from being well characterized. Autopsies, although not sufficient in number to reach conclusive results, have furnished important and unexpected information [52]. For example, in lung lesions, a high percentage of small thrombi have been identified in the small arterial vessels that often include more than 25% of the lung tissue, thus explaining the severe hypoxemia characteristic of Covid-19 patients. Microthrombi are often found in the region of alveolar damage along with endothelial damage [52,53]. This underlines an extremely relevant activation of the coagulation process in the acute respiratory distress syndrome (ARDS) and in the high mortality rate of Covid-19.

Since SARS-CoV-2 targets preferentially ACE2-expressing cells and numerous cell types express this receptor, the large distribution of ACE2 might be a primary cause of the multiorgan disease. However, the complex maturation mechanism of the spike protein could also facilitate the invasion of ACE2-negative cells and activate a more intricate relationship with the immune system and the cytokine storm activation. In hospitalized Covid-19 patients, ARDS usually coincides with the viral load peak about eight days after the symptom onset, suggesting that more waves of viral load/spread can occur [6,29]. In addition, the highest viral load might coexist with secondary bacterial and fungal infections that might be most probably involved in lung injury through the stimulation of the cytokine storm [6,29]. The cytokine storm is frequently described as induced by other viruses or bacteria and as a critical trigger for the activation of the coagulation pathways [53]. The imbalance between direct viral targeting and the immunopathogenic response in lungs promotes ALI and the systemic complications. Thus, the comprehension of the pathophysiological mechanism of Covid-19 is made complex by the intersection of various phenomena of variable entity. Their characterization is absolutely mandatory for the development of effective pharmacological and immunologically-based therapies.

While the key importance of DCs in the innate and adaptive immune response in infectious diseases has been conclusively demonstrated, the specific role of DCs in the Covid-19 pathology has been insufficiently studied, so far. However, several direct and indirect pieces of evidence suggest that these cells might be involved in the development and evolution of the disease, although it is unclear whether DCs might be effectors of SARS-CoV-2 activity, targets of virus infections, or both.

A relative increase of mature DCs in patients’ BALs (broncho-alveolar lavages) has already been reported, suggesting that these cells participate to the lung response in SARS-CoV-2 infection [54].

Autopsies also demonstrated modification of lymph nodes and spleen, with frequent signs of atrophy. It is however important to underline that the number of autopsies performed by each center is still limited and the authors of the autopsy studies correctly discussed this bias.

Accurate investigations have demonstrated that DCs play an important role in the two previous human coronavirus outbreaks. In both diseases, like Covid-19, clinical manifestation includes a rapid and progressive acute pneumonia with altered multiorgan functions. MERS-CoV has been demonstrated to infect Mo-DCs, rapidly inducing high expression levels of IFN-γ, IP-10, IL-12 and RANTES (regulated on activation, normal T cell expressed and secreted). The infection results in no IFN-β expression and only marginal IFN-α production [55]. Importantly, MERS-CoV requires dipeptidyl peptidase 4 (DPP4) receptor for intracellular entry and this protein is strongly expressed in DCs cells [56]. Moreover, DC infection by MERS-CoV has been demonstrated to be productive [45].

The mechanism of SARS-CoV access into DCs is mostly debated. However, the entry is demonstrated to occur by the DC production of pro-inflammatory cytokine TNF-α and IL-6, as in MERS. As for MERS-CoV, no interferon production has been reported [45]. Although DCs express intermediate levels of ACE2 (lower than epithelial/endothelial cells and higher than lymphocytes), the entry could be due to micropinocytosis or DPP4 interaction. Moreover, and more interestingly, several studies have demonstrated the ability of SARS-CoV to use DC-SIGN (see next paragraph for DC-SIGN detailed description) as an alternative receptor or as a factor facilitating ACE2-mediated virus infection [57,58,59]. DC-SIGN might serve as receptor independently of ACE2 and no synergy occurs between the two receptors [55]. Mechanistically, glycan levels on the spike protein are essential for the binding to DC-SIGN. Finally, the glycosylated arginine residues involved in the interaction with DC-SIGN are distinct from those involved in ACE-2 binding [59]. In addition to the in vitro studies, the spatio-temporal evolution of SARS-CoV infection has been investigated in rhesus macaques. In this study, it was shown that, as expected, innate immunity plays a major role in early virus replication and dissemination. However, it has also been shown that monocytes migrate into tonsils and draining lymph nodes within few days. Monocytes, upon differentiation into DCs, provide a systemic dissemination of virus after 3 days from infection [60]. It is important to emphasize once again that human SARS-CoV and SARS-CoV-2 generally employ ACE2 for cell entry and thus a comparison between the two coronaviruses is particular relevant and worthwhile.

Three very recent investigations directly describe a role of DC cells in Covid-19 disease. First, Sanchez-Cerrillo and colleagues analyzed the distribution of DC subsets in the lungs and blood of patients and found that circulating CD1c+, CD141+ and pDCs diminished. Importantly, while CD1c+ accumulated in the lung, this was not observed in the case of CD141+ and pDCs, indicating a general decrease of these DC subsets [61]. Subsequently, Zhou et al. studied a cohort of 17 acute and 24 convalescent patients, observing a significant reduction of blood DC cells with an impairment of their activity. In addition, a large increase of the cDC/pDC ratio was observed in severe acute patients. The authors suggested that the specific diminution of pDCs could explain the reduction of the interferon production and the early decrease of innate immunity against SARS-CoV-2 infection. Complex alterations of adaptive immunity were also highlighted, which could be, at least in part, due to the DC compromission. Notably, while neutralizing antibodies against RBD and NP were produced in abundance in all patients, T cell response was remarkably delayed and due essentially to CD4+ T-cells. This in turn, might strongly influence vaccine efficacy being CD8+ T cells important constituents in immune memory maintenance [62]. A third study, performed by Yang et al., confirmed that SARS-CoV-2 might enter DCs and macrophages but, as in the case of SARS-CoV, without a permissive replication. The entry process has also been proposed to occur through DC-SIGN and furin and not through ACE2 and TMPRSS2 [63]. Finally, there are no data on antigen processing and presentation by DCs during Covid-19 infection. However, at least in the case of SARS-CoV, infected DCs do not mature to perform direct antigen presentation to activate T cells [64].

In brief, all these recent data strongly argue in favor of the involvement of DCs in Covid-19 disease. Moreover, whether the negative effects of SARS-CoV-2 infection on DC population will be definitely confirmed, this could be a major negative factor in vaccine development. The reported reduction of DC function also suggests that strategies for reverting this negative effect might be useful in Covid-19 therapy. DC numbers could be increased by various treatments (namely, systemic injection of TLR7 agonist, administration of Flt3L, Flt3L plus TLR agonists or STING agonists). However, it remains to be seen whether these approaches might be successful. Alternatively, DCs could be isolated from patient’s peripheral blood, loaded with antigens, and matured using various cytokine cocktails (like GM-CSF/prostatic acid phosphatase fusion protein). However, so far, ex vivo dendritic manipulation does not seem very effective.

In the next paragraphs, we will discuss various mechanisms by which SARS-CoV-2 might affect DC activity damaging their physiological role in immune response. Initially, we will analyze the possible role of DC-SIGN in SARS-CoV-2 outbreak. A second process that could affect DC activity is related to the interactions of the virus with the pathways regulated by ACE2. Finally, we will discuss the effects of Covid-19-dependent hypercoagulability on DC activity.

## 7. DC-SIGN and Sars-CoV-2 Infection

DCs are the most powerful professional antigen presenting cells. They have a peculiar characteristic of being able to modulate positively or negatively (i.e., suppress) the immune system. DC-specific molecular nonintegrin-3-adherent (DC-SIGN, CD209) is a member of the superfamily receptor of type C lectin which is highly expressed on the DC surface [65]. DC-SIGN allows DC to interact with both pathogens and endothelial, epithelial and myeloid cells. The binding requires mannose residues on pathogens or fucosylated residues on cell membranes [66]. From a structural point of view, DC-SIGN is a transmembrane protein (404 amino acids) which, like many other receptors, includes a short cytosolic region, a transmembrane domain and a long extracellular region (called the extended extracellular domain, ECD). The ECD consists of two functionally distinct domains, namely, a neck sequence (containing seven and a half repetitions of 23 amino acid residues) and a domain capable of recognizing carbohydrates (calcium-dependent carbohydrate recognition, CRD). Intriguingly, the affinity of sugars for DC-SIGN CRD is low (with an affinity constant, Kd, in the mM range).

However, the neck domain allows an oligomerization process resulting into a tetrameric form that recognizes heavily-glycosylated ligands with high affinity and avidity (Kd in nM range) [67]. DC-SIGN is expressed in some specific DC populations, and in minor amounts, in B-cells and macrophages. Specifically, DC-SIGN occurs in dermal and stromal DCs but scarcely in follicular DCs. It is poorly expressed in monocytes but increases extremely when monocytes leave the blood and differentiate into DCs. When DCs migrate to secondary lymphoid tissues, a decrease of DC-SIGN expression occurs. The activation of DCs is a major mechanism of regulation of DC functions such as adhesion, migration, inflammation, T cell activation and induction of immune response.

Functionally, DC-SIGN is specialized in the recognition of viral glycosylated proteins with high mannose expression. So far, the main role of DC-SIGN has been reported in the process of trans-infection, described in HIV-1 infection and transmission [66]. In particular, the DC-SIGN signaling activation on DCs induces their migration in lymph nodes where the receptor promotes the transfer of intact HIV-1 virus from DCs to CD4 T-cell, facilitating T-cell infection and viral spreading. This process is controlled by at least two critical points: DC-SIGN-pathogens binding on DCs membrane and the following viral internalization [66,67,68].

DC-SIGN receptor includes a C-terminal carbohydrate-recognition domain that is involved in the interaction with glycoproteins expressed in the viral envelope, whereas the N-terminal cytoplasmic domain shows a LL (leucine-leucine) motif that mediates the ligand internalization. The presence of LL-motif has a central function in trans-infection process. Indeed, its removal lowers the HIV-1 transmission efficiency. Interestingly, a reduction of the intracellular pH promotes viral internalization and, then, transmission induced by DC-SIGN pathway [69]. In addition, it has been hypothesized that a low pH could alter the glycosylation levels in the viral envelope, thus, increasing the infectivity related to the DC-SIGN activation [57,69].

DC-SIGN receptors are expressed in both immature and mature DCs. However, the activation of DC-SIGN pathway has shown to down-regulate the cellular production of histocompatibility complex class II that has a significant role in antigen-presenting activity [44]. The HIV-1-DC-SIGN binding also promotes the Th2 response reducing the production of IL-2 and the stimulation of interferons response [64]. Then, it favors the migration of macrophages and others DCs releasing chemokines such as MIP-1α and RANTES. Furthermore, the recruitment of these cells induces the secretion of inflammatory cytokines such as IL-6 that upregulates DC-SIGN expression in vivo [64]. Accordingly, either mature or immature DCs may represent the “Trojan horse” in HIV-1 infection and could have a remarkable impact on the inflammatory response stimulated by these cells. Furthermore, it has also been reported that the interaction of DC-SIGN with HIV gp120 induces DC apoptosis. This indicates that the effect of DC-SIGN stimulation on DC phenotypes and survival might vary in a context-dependent way.

HIV-1 does not represent the only virus that enhances its infectivity through DC-SIGN binding. Other viruses use this molecular signaling pathway, especially Cytomegalovirus (CMV) and Ebola virus [69,70]. CMV interaction with DC-SIGN is mediated by its envelope glycosylated protein CMV gB (glycoprotein B) and this event induces the virus transmission to T-cell and subsequent spreading, in the same way as HIV-1 [71]. Mechanistically, DCs have been shown to transmit HIV-1 and CMV to other cells through DC-SIGN receptor after five days in cultured cells. Thus, it has been postulated that DCs may transport some viral particles for a long period and, thus, could rekindle the infection later. Ebola virus has been shown to promote susceptibility to the infection through binding to DC-SIGN through its glycosylated protein GP expressed in its envelope [69].

All these data suggest that the role of DC-SIGN in Covid-19 infection should not be overlooked but needs to be taken in consideration. The possibility that DCs, by DC-SIGN, might be involved in SARS-CoV-2 infection and Covid-19 disease is based on previous data reported in studies on the role of DCs in human coronavirus infections. In particular, in MERS-CoV and SARS-CoV infections, a possible role of DC-SIGN has been suggested [57,58]. However, a major difference between MERS-CoV and SARS-CoV appears to be the productivity of the viral infection. On the other hand, even in the absence of a productive infection, DCs can represent a mechanism of spreading of (inactive or already pre-activated) virions or a reservoir for viral particles [57,64,72,73]. Alternatively, it is possible that the interaction of the virus or its envelope proteins (including products of pyroptosis) with DC-SIGN results in a loss of DC population or of a specific DC subpopulation.

An intriguing and additional aspect, scarcely discussed in literature, regards the mutations of the spike protein already demonstrated. Specifically, changes in the glycoprotein S of SARS-CoV-2 affecting its glycosylation may modify the virulence. As a matter of facts, S is a heavily glycosylated protein. An increased level of glycosylation in this protein might result in a higher virulence compared to parental virus strain, which could be ascribed to a possible enhancement in the affinity between spike and its receptor ACE2. However, higher levels of glycosylation may also stimulate DC-SIGN binding (with a greatest affinity) and signaling [12]. DC-SIGN may be involved in Covid-19 pathogenesis and the evaluation of this mechanism could have a pivotal role in the identification of new therapeutic strategies and development of a vaccine. Indeed, the SARS-CoV-2/DC-SIGN interaction on the membrane surface of macrophages and, mostly, of DCs may activate multiple escape mechanisms.

A final consideration could be made that refers to the molecular hiding of SARS-CoV-2 in DCs. This could affect T-cells activity at lymph node level, preventing their recruitment at the inflammatory site. The detrimental effects on T-cell population (or subpopulations, particularly CD8+) could also generate alteration in the memory cells with a negative impact on vaccine efficacy.

## 8. RAAS Dysregulation, Aldosterone and Dendritic Cells

In 2003, the ACE2 protein was identified by immunoprecipitation experiments and mass spectrometry analyses as the cell membrane interactor of SARS-CoV S protein [74,75]. ACE2 has also been demonstrated as the primary receptor of SARS-CoV-2 [76]. The finding functionally correlated Covid-19 disease with the renin-angiotensin-aldosterone system (RAAS) and suggested to evaluate the effect of ACE inhibitors and angiotensin receptor blockers (ARBs) on the ongoing ruinous outbreak. Although not been fully established, broad consensus now demonstrates that no negative relationship exists between the use of ACEIs/ARBs and evolution of Covid-19 infection [77,78,79,80].

ACE and ACE2 show an opposite role in the control of plasma volume. In brief, a reduction of kidney blood flow causes, in juxtaglomerular cells, the conversion of prorenin into renin. Subsequently, plasma renin catalyzes the formation of angiotensin I from angiotensinogen. Angiotensin I (Ang 1, a decapeptide) is then converted into angiotensin II (Ang II, an octapeptide) by ACE [81], which is localized at the membrane level of endothelial cells, particularly on the vasculature of lung. Finally, Ang II is converted into a smaller peptide Ang(1–7) by ACE2 localized on the membrane of numerous different tissues. While Ang I does not appear to have biological function, Ang II exerts several important functions acting on two different receptors, namely, AT1 and AT2. The activation of AT1 results in vasoconstriction, inflammation, insulin resistance, atherogenesis and thrombosis. Ang II, acting via AT1, stimulates the synthesis of aldosterone, which might be considered the late step of the RAAS pathway. AT2 receptor is scarcely expressed in adult tissues. After its stimulation, vasodilatation, insulin activation and reduced platelets aggregation are observed. Ang(1–7), that exerts its activity by MAS-related G-protein coupled receptor, has been shown to possess anti-inflammatory effects and anti-oxidant properties [81,82]. In blood, it induces vasodilatation by causing the release of prostanoids and nitric oxide [81,82,83].

Ang II and aldosterone are tightly involved in cardiovascular remodeling and fibrosis and also have systemic effects inducing vasoconstriction, inflammation and oxidative stress [84]. SARS-CoV-2 interaction with ACE2 results in a quantitative decrease of the enzyme [76,85]. In turn, this results in Ang II up-regulation and stimulation of RAAS pathway. The mechanism has been demonstrated in patients by the correlation between high Ang II levels, viral load and lung injury in Covid-19 pneumonia [76,86]. A similar effect has been demonstrated in cell lines infected with SARS-CoV [45]. Furthermore, conditions associated with overactivation of RAAS such as hypertension, diabetes and cardiovascular diseases have shown the highest rate of intensive care unit hospitalization and mortality [87]. It has been proposed that Covid-19-dependent Ang II accumulation promotes ALI through the activation of inflammation (due to the cytokine storm), activation of NADH/NADPH oxidase system and vasoconstriction [76,86,88]. These detrimental effects could be also mediated by aldosterone [84,89,90]. High levels of Ang II are the main stimulator of aldosterone in the adrenal cortex. Indeed, the activation of mineralcorticoid receptor (MR) results in systemic effects similar to those due to Ang II. Importantly, MR occurs in several tissues, including heart, kidney, endothelial and immune cells [84]. High levels of aldosterone are described in subjects affected by cardiovascular diseases (hypertension, ischemic cardiomyopathy and heart failure) and they might persist in these patients even under ACEIs/ARBs therapy [91]. Conversely, plasma aldosterone returns to pretreatment levels in up to 30–40% of patients on oral ACEIs and ARBs therapy through the phenomenon of “aldosterone breakthrough” [92].

The role of aldosterone, however, appears underestimated in SARS-CoV-2 infection. The hormone may be involved in severe lung injury induced by Covid-19 with an Ang II similar mechanism, in other words, activating the proinflammatory pathway [90] (see Figure 2).

It induces activation/migration of immune cells and, particularly of macrophages, by stimulating the expression of cyclooxygenase-2 and intercellular adhesion molecule-1. In addition, MRs are expressed in DCs and their activation might stimulate the secretion of IL-6 and TGF-β1 [84]. DCs seem to be an immunological target of aldosterone, and the modulation of this cell phenotype might be of relevance in the control of T-cell-dependent adaptive immune response. Thus, aldosterone may induce severe lung injury in patients with Covid-19 pneumonia stimulating a greater inflammatory response and cytokine storm through the activation of DCs. The effect on the production of IL-6 appears of peculiar interest [93]. Accordingly, clinical trials are evaluating the role of IL-6 receptor antagonists, like tocilizumab, in treatment of patients with Covid-19 pneumonia and cytokine release syndrome [42]. It is also possible that the DCs stimulation by Ang II/aldosterone together with the direct effects of the virus on this population (see previous paragraph) can alter the proliferation and maturation of DCs with consequent functional dysregulation of these cells.

The role of aldosterone in ALI is not yet defined and the mechanism of production and activation of this hormone in the lungs is not clear. A comparison with SARS-CoV infection might help. SARS-CoV has been shown to reduce ACE2 in lung cells and, in turn, stimulate local Ang II production. This, in turn, induces its detrimental effect by binding AT1 receptors primarily on endothelial cells [94]. The increase in Ang II also promotes cell damage and pyroptosis in pneumocytes and stimulates the secretion of aldosterone by the adrenal cortex. Ang II and aldosterone can synergistically stimulate the systemic and local inflammatory pathway in the lungs. Furthermore, local activation of Ang II in lung cells can induce direct production of aldosterone in the lungs by upregulating aldosterone synthase in endothelial cells [84,95].

In conclusion, the overactivation of the RAAS system induced by Covid-19 with high levels of Ang II and aldosterone may have a role in the modulation of DCs activity in patients with pneumonia.

## 9. Dendritic Cell and Hypercoagulability

A procoagulant state is a critical condition in patients with Covid-19 pneumonia [53,96]. In this disease, hypercoagulability is associated with higher mortality and elevate levels of D-dimer correlate with the worst prognosis [97]. Accordingly, autopsy studies have reported the frequent presence of microthrombi, especially in patients with sepsis and multiorgan failure [52]. In Covid-19 pneumonia, the rapid viral diffusion and cytokine storm induced by SARS-CoV-2 may be the primary trigger of coagulopathy [98]. As previously reported, ACE2 is strongly expressed in several tissues/cells, including lung, kidney, heart, intestine and endothelial cells [76]. However, the presence of ACE2 on the membrane is not sufficient to demonstrate virus entry since several additional events are required (see above). Direct confirmation of endothelial cell invasion came from investigations that reported that SARS-CoV-2 could infect blood vessel organoids [99]. These studies allow the view that the virus is incorporated into endothelial cells. More direct evidence has been obtained by electron microscopy analyses of endothelial cells of glomerular capillary loops. Viral particles have been indeed detected in these cells [32].

Thus, coronavirus entry into endothelial cells promotes cytotoxic effects/pyroptosis, induces microvascular dysfunction and activation of inflammatory pathway in vessels. Moreover, vascular injury causes the activation of thrombin with a consequent procoagulant state and, central for the diseases, the viral diffusion in blood. In brief, Covid-19 endotheliitis represents the most important trigger for sepsis. The inflammation status and hypercoagulability are also tightly connected in that the procoagulant state shows a positive synergism with the inflammatory response. In particular, the coagulation pathway may directly stimulate cytokine storm by activating proteinase-activated receptor (PARs) in Covid-19 [53,98]. Member of PAR family, namely, PAR-1 and PAR-2, are receptors activated by thrombin. In murine models, PAR-1 inhibitions reduced neutrophilic lung inflammation, cytokines response and lipopolysaccharide-induced lung injury. PARs have also shown similar proinflammatory mechanisms in infection induced by coxsackievirus and influenza A virus [53,98,100].

In endothelial cells, the enhanced thrombin-PAR-1 signaling also induces the migration and the activation of DCs that further stimulate the systemic inflammation and coagulation [101]. This amplificatory loop has also shown a significative role in sepsis progression. Moreover, the occurrence of an amplificatory loop strongly suggests an involvement of DCs in the hypercoagulability state in Covid-19 pneumonia. DC activation, induced by thrombin-PAR-1 interaction, might be also involved in a second mechanism associated to RAAS overactivation. As already described, RAAS overstimulation and PAR-1 activation promote endothelial damage. In particular, PAR-1 stimulates aldosterone secretion, as demonstrated in mice [102] and increases the Ang-II-associated remodeling of cardiovascular system. Ang II might stimulate the procoagulant state, mostly through two possible mechanisms. First, it up-regulates tissue factor and levels of plasminogen activator inhibitor 1. Second, it reduces antithrombotic proteins as antithrombin III [103,104].

A number of findings suggest a positive feedback between the RAAS pathway and PARs activation. In particular, Ang II increase (observed during SARS-CoV-2 infection) upregulates PAR1 mRNA expression, potentiating the effect of thrombin on the vascular wall and the inflammatory response [105]. Thus, thrombin-PAR-1 signaling may activate DCs’ response through a second mechanism dependent on the increased levels of Ang II and, mostly, of aldosterone. On the other hand, the activation of DC linked to inflammation and coagulation might have an additional consequences in that it can decrease circulating DCs due to their tissue relocalization similarly to what has been demonstrated for DC1c+ accumulation in the lung.

In summary, DC activation may be strongly involved in hypercoagulability induced by Covid-19 pneumonia. As a consequence of the altered coagulability and increased inflammation, prophylactic doses of low molecular weight heparin are employed in Covid-19 patients.

## 10. Conclusions

SARS-CoV-2 is the causative agent of Covid-19 outbreak and poses a terrible threat to global health. At the time of preparation of this review (27 August 2020) more than 2.42 × 10^7^ subjects have been infected and the epidemic is active in many areas of world. Furthermore, it is not clear yet whether the peak of the infection has been reached or if the pandemic is still in an exponential growth phase. One of the most important strategies for disease containment and prevention is considered the development of vaccine(s) able to induce an immune response against viral proteins that play a key role in the infection. Mechanistically, the primary target is believed to be the S protein which is required for cell target identification and, subsequently, virus entry. Several studies have investigated the mechanisms of S protein function in detail, including the complex process of its maturation. However, a definite framework is still incomplete and further studies are clearly needed. Particularly, the identification of different proteases involved in S protein cleavage/activation as well as the importance of alternative cellular targets and the role of glycosylation in forming a shield on the spike structure have not been established.

On the other hand, the development of an efficacious immune response involves several cell players, a large number of cytokines and a well-orchestrated sequence of events. A precise definition of their coordination is absolutely necessary also in view of the onset of novel SARS-CoV-2 mutants or possible new epidemic zoonoses. In particular, the emergence of mutants is a highly probable event considering the large number of infected subjects and the consequent enhanced possibility of recombination. At the moment, few new virus variants have been identified, that, fortunately, although apparently more contagious, do not show an increase of mortality rate. However, as for other viruses, changes in S sequence or glycosylation might affect the process of target (i.e., the membrane receptor) identification and vaccine efficacy. Virus evolution and host adaptation are well known processes not completely clarified, although studied in detail in influenza pandemics.

A pivotal role in both native and adaptive immune response is played by DCs. Their importance in the Covid-19 disease has not been well defined due to the strong alterations observed in other cell populations of innate immunity. However, the complete remodeling of the immune response following Covid-19 as well as the function played by DCs in SARS-CoV and MERS-CoV infections suggest a possible function in virus spread, hypercoagulability, inflammatory response and multiorgan dysfunction. Additionally, recent findings suggest Covid-19 patients have a decrease of circulating pDC and CD141+ due to a not clearly defined mechanism [62]. DC reduction might have important negative consequence on T-cell immune memory maintenance and vaccine preparation,

Ex vivo studies, although fundamental in understanding the mechanism of infection, do not clarify the disease pathophysiology at an integrative level. For example, it has been reported that Mo-DCs are able to interact with SARS-CoV and SARS-CoV-2 but (differently from MERS-CoV infection) the interaction is not productive [45]. However, the virus might be sequestered in intracellular vesicles and consequently it can be spread throughout the human host. This is functionally confirmed by data in animal models of SARS-CoV infection that undoubtedly suggest a key role of Mo- DCs in SARS-CoV spread [60]. These results indicate that further studies, conducted in integrated model systems, on the relationships among infection mechanisms, cellular interactions, virus load and spread, multiorgan failure, inflammation and hypercoagulation are absolutely necessary.

Finally, an additional point will merit investigation in the context of DC’s role in SARS-CoV-2 infection. In particular, the importance of the reported ability of estrogen to stimulate DC activity [106] needs explaining and clarifying as to why women appear less likely to die from Covid-19 than men.

We are still long way from understanding the interaction between coronavirus infection and the immune system function, and still longer will be taken to effectively address potential future waves of the viral pandemic and to develop therapeutic challenges for their treatment.
